# Role of *OPRM1*, clinical and anthropometric variants in neonatal pain reduction

**DOI:** 10.1038/s41598-020-63790-2

**Published:** 2020-04-27

**Authors:** Ilaria Erbi, Massimiliano Ciantelli, Riccardo Farinella, Cristina Tuoni, Manuel Gentiluomo, Francesca Moscuzza, Cosmeri Rizzato, Alice Bedini, Maddalena Faraoni, Stefano Giusfredi, Arianna Tavanti, Paolo Ghirri, Daniele Campa

**Affiliations:** 1 Department of Biology, University of Pisa, Pisa, Italy; 2 Division of Neonatology – Santa Chiara Hospital, Pisa, Italy; 3 Department of Translation Research and of New Technologies in Medicine and Surgery, University of Pisa, Pisa, Italy

**Keywords:** Genetic association study, Pharmacogenomics

## Abstract

An increased awareness on neonatal pain-associated complications has led to the development of pain scales adequate to assess the level of pain experienced by newborns such as the ABC score. A commonly used analgesic procedure is to administer a 33% oral dextrose solution to newborns prior to the painful intervention. Although this procedure is very successful, not in all subjects it reaches complete efficacy. A possible explanation for the different response to the treatment could be genetic variability. We have investigated the genetic variability of the *OPRM1* gene in 1077 newborns in relation to non-pharmacologic pain relief treatment. We observed that the procedure was successful in 966 individuals and there was no association between the genotypes and the analgesic efficacy when comparing individuals that had an ABC score = 0 and ABC score >0. However, considering only the individuals with ABC score>0, we found that the homozygous carriers of the G allele of the missense variant SNP rs1799971 (A118G) showed an interesting association with higher ABC score. We also observed that individuals fed with formula milk were more likely to not respond to the analgesic treatment compared to those that had been breastfed.

## Introduction

Until few decades ago, it was thought that newborns were unable to feel pain: for this reason, many painful procedures, such as surgical interventions, tracheal intubation or venipuncture, were performed without analgesia. However, there are overwhelming evidences supporting the fact that the ability to respond to painful stimuli starts during intrauterine life^1–6^. Pain related stress has been associated with poor growth and neurocognitive impairment in term and preterm infants^3,5–10^. Intracranial hemorrhage and periventricular leukomalacia have been described as short term complications of painful procedures while behavioral disorders, anxiety spectrum disorders, sleep disorders, reduced post-natal growth and poor neurological outcome have been identified as long-term complications of prolonged stress/pain in early life^8,9^. An increased awareness on neonatal pain-associated complications has led to the development of pain scales adequate to assess the level of pain experienced by newborns (ABC scale, PIPP scale). These scales are based on behavioral changes (crying, changes in facial expression) and vital signs (heart rate, respiratory rate) during painful procedures. All term infants are commonly subjected to painful procedures before discharge at home for neonatal rare disease screening purposes; preterm newborns experience more invasive procedures such as intubation, central vein catheterization and may undergo 3 or 4 blood sampling every day for the first few weeks of life. Alongside improved methods to assess pain, also clinical procedures to alleviate it have been developed. With this regard, for major painful procedures, such as tracheal intubation, opioids and benzodiazepines are recommended, while less painful procedures (e.g. venipuncture or capillary blood sampling from the heel) are generally performed under a non-pharmacological analgesia. A commonly used approach is to administer an oral dextrose solution (20 to 33% concentration) to newborns prior to the painful intervention^11^. Although this procedure is very successful, not in all subjects it reaches analgesic efficacy. Numerous evidences suggest the involvement of the mu opioid receptor (MOR-1) in the analgesic efficacy of the dextrose solution. However, it is not clear if this effect is achieved through a direct interaction between the sugar and the receptor or through the regulation of endogenous opioids. Taddio and colleagues in a very small study consisting in 11 preterm infants aimed at establishing a direct link between beta-endorphin increase after sucrose administration, did not report a statistically significant association^12^. However, several studies conducted using animal models suggest the release of endogenous opioids through the blockage of mu opioid receptor during the administration of sweet substances^13^. To support this hypothesis, there is also the observation that the greatest analgesic efficacy is recorded after about two minutes from the beginning of glucose administration, time lapse that coincides with that necessary for the release of endorphins^14^. An additional indirect association between the MOR-1 receptor and dextrose/sucrose analgesia is the observation that newborns of methadone addicted mothers did not respond to orogustatory (sucrose) stimulation^15^. Finally, the analgesic effect of oral dextrose may also be attributable to an increase in plasma insulin levels which in turn has been shown to have analgesic activity through the regulation of many pathways^16,17^. The MOR-1 receptor is encoded by the *OPRM1* gene that is highly polymorphic and many studies performed in adults have suggested an association between the genetic variability in the *OPRM1* gene, and the response to pain relief treatment in adults^18–25^. Despite all these evidences, to the best of our knowledge, there are no studies linking the effect of the dextrose/glucose treatment with the genetic variability of the gene. With these premises we have investigated for the first time the genetic variability of the *OPRM1* gene in 1077 newborns collected at University Hospital of Santa Chiara, in relation to non-pharmacologic pain relief treatment.

## Materials and methods

### Study population

Blood samples from 1077 neonates born between 2015 and 2019 were collected at the Division of Neonatology of the Santa Chiara Hospital. For each newborn 5 ml of blood were collected from the cord at birth, in a completely not invasive way. Anthropometric measures at birth (birth weight, length, head circumference), type of feeding (exclusive breastfeeding, partial breastfeeding and exclusive formula milk) data on delivery (spontaneous vs caesarean section and mother’s pharmacological analgesia if present) and familiar history (ethnicity, mother’s age, pre-pregnancy BMI, weight increase during pregnancy, relevant diseases) were also collected. The parents of all subjects signed a written informed consent form the study was conducted in accordance with the Declaration of Helsinki and was approved by the ethical committee of the Meyer Children Hospital of Florence.

### Pain relief determination

In order to reduce variability secondary to different type of procedures, we assessed just heel lancing for neonatal metabolic screening. According to Italian Neonatal Society guidelines^26^ and international guidelines for pain relief in newborns, before the procedure to all neonates a 33% dextrose solution was orally administered in order to reduce pain perception. The heel incision device used was Gentle heel™ produced by Alleset, Inc (Flowery Branch, USA). Pain level was assessed with ABC scale, which consists of three cry parameters: (A) First cry acuteness (NO = 0; YES = 2), (B) Burst rhythmicity (NO = 0; YES = 2), (C) Cry constancy (no cry or only a brief moan = 0; not constant, but more than a brief moan = 1; constant = 2). The ABC scale was validated for healthy, non-intubated term newborns^27^. Clinical procedures and pain recording were carried out at the Neonatology Unit of the Santa Chiara Hospital by trained personnel only.

### SNPs selection

Common genetic variability in the *OPRM1* gene region was investigated following a hybrid functional and tagging approach to identify candidate SNPs. For *OPRM1* tagging SNPs were selected with the use of the Haploview Tagger Program (http://www.broad.mit.edu/mpg/haploview/; http://www.broad.mit.edu/mpg/tagger/)^28^, using pairwise tagging with a minimum r^2^ of 0.8. In addition, we have included in the selection the *OPRM1*-rs1799971 (A118G) that is a putatively functional SNP. The final selection included 11 SNPs for the *OPRM1* gene.

### DNA extraction and genotyping

DNA was extracted from umbilical cord blood using Quick-DNA Plus Kit (Zymo Research). Genotyping was performed using the allele-specific TaqMan PCR SNP genotyping assay (Thermo Fisher Scientific, Waltham, Massachusetts, USA) as recommended by the manufacturer. Detection of the genotyping calls was made using the QuantStudio 5 Real-Time PCR System (Applied Biosystems by Thermo Fisher Scientific, Waltham, Massachusetts, USA), 3.5% of the samples were duplicated to ensure genotyping quality.

### Statistical analysis

Hardy-Weinberg equilibrium was tested by the chi square test. The association between the SNPs, the covariates considered (mentioned before) and pain relief treatment was calculated using an unconditional logistic regression computing Odds Ratio (OR) and confidence intervals (CI) considering the ABC score as a categorical variable (ABC score = 0 *Vs* ABC score >0). In addition, we also performed an analysis considering only the individuals that had and ABC score>0 and calculated the association between the genotypes and the ABC score (ABC score coded as 1;2;3;4;5 and 6) with a general linear model (glm). Genetic analyses were performed under a co-dominant inheritance model. For a subgroup of individuals (n = 845), we have also collected data on the person performing the sedation (so forth called operator) and we used this variable for adjustment. We performed crude analysis (without adjusting for operator) and adjusted analysis. The glm model was adjusted for gestational age and operator, since these variables were the only ones showing a borderline association with the ABC score.

### Bioinformatic analysis

We used several bioinformatic tools to assess possible functional relevance for the SNPs showing significant associations. RegulomeDB (http://regulome.stanford.edu/)and HaploReg^29^ were used to identify the regulatory potential of the SNPs, The Genotype-Tissue Expression (GTEx)^30^ was used to identify potential associations between the SNP and expression levels of nearby genes (eQTL).

## Results

### Data filtering and quality control

All SNPs genotype distribution were in Hardy-Weinberg equilibrium with a P-value > 0.005. The average polymorphism call rate was 98.84% with a minimum of 97.62% for rs610231 and a maximum of 99.72% for rs2075572. The concordance rate for the duplicated samples was more than 99%.

### Anthropometric, clinical and lifestyle variables and analgesic treatment response

Oral dextrose administration was successful in avoiding pain related to minor painful procedures in 966 individuals out of 1054; in 88 patients it was not effective: 9 had an ABC score of 1, 40 of 2, 13 of 3, 17 of 5 and 9 of 6. For 33 individuals the ABC score was not calculated. In the crude analysis we observed that the type of feeding (exclusive breastfeeding, partial breastfeeding and exclusive formula milk) had an effect on the ABC score with the tendency (P test for trend p = 0.005) of mixed types and artificial feeding to increase the chance of having an ABC score > 0 with OR 1.90 (95% CI 1.18–3.06; P-value = 0.008) for mixed type and OR 2.23 (95% CI 0.88–5.64; P-value = 0.088) for formula milk. In addition, we observed that also the age of the mother had a weak effect on the ABC score with OR 1.05 (95% CI 1.01–1.09; p = 0.036) for each year increase in the maternal age. These results are shown in Table 1. The other variables taken into consideration did not show any significant result. Adjusting for operator we observed similar results (Supplementary Table 1). Considering the subjects with ABC score > 0 none of the variables showed a statistically significant association, with gestational age and operator showing a borderline association with the ABC score (Supplementary Table 2).

**Table 1:** Logistic analysis between Anthropometric, clinical and life style variables and ABC score.

Covariates	Logistic analysis		
	OR^(a)^(CI)^(b)^	p-value	p-trend
Gender_(m/f)	0.87(0.56–1.34)	0.521	0.520
Gestational age_(weeks)	0.99(0.83–1.19)	0.939	0.939
Procedure’s executor	1.03(1.01–1.05)	0.005	0.005
Feeding type_(maternal/ mixed)	1.90(1.18–3.06)	**0.008**	0.005
Feeding type_(maternal/ artificial)	2.23(0.88–5.64)	0.088	
Mode of birth_(vaginal delivery/ cesarean section)	1.07(0.69–1.66)	0.757	0.757
Maternal age_(years)	1.05(1.01–1.09)	0.036	0.036
Spinal anesthesia_(yes/no)	1.19(0.75–1.89)	0.468	0.468
Epidural anesthesia_(yes/no)	0.83(0.45–1.51)	0.534	0.534
General anesthesia_(yes/no)	1.35(0.17–10.76)	0.775	0.774
Birth weight_(gramms)	0.99(0.99–1.00)	0.118	0.118
Maternal gestational diabetes_(yes/no)	1.16(0.65–2.08)	0.616	0.616
Mother’s smoke_(yes/no)	1.64(0.68–3.98)	0.274	0.270

^(a)^OR identifies the Odds Ratio.

^(b)^CI represents the Confidence Interval.

### SNPs effect on analgesic treatment

In the crude analysis we observed no statistically significant association as shown in Supplementary Table 3. Adjusting for operator we observed that rs510769 was close to the conventional threshold for statistical significance in the codominant model: OR 1.6 (95% CI 0.99–2.58) p = 0.055 as shown in Table 2. Considering the subgroup analysis of the individuals with an ABC score > 0, we observed that homozygous carriers of the G allele of the missense SNP rs1799971 (A118G) were associated with a higher ABC score, although the results were borderline not significant (p = 0.055) as shown in Supplementary Table 4. We also performed exploratory analysis adjusting for gestational age as shown in Table 3, operator (Supplementary Table 5) and gestational age and operator (Supplementary Table 6). *OPRM1*-rs1799971 (A118G) was associated with high ABC score reaching statistical significance when adjusting for gestational age (p = 0.041).

**Table 2:** Association between OPRM1 polymorphisms and ABC score (ABC score > 0 vs ABC score = 0).

SNP	ALLELES	ABC SCORE > 0^(a)^			ABC SCORE = 0^(b)^			Codominant- heterozygous^(c)^		Codominant- Recessive ^(d)^		Dominant^(e)^		Recessive ^(f)^	
		MM^(a)^	Mm^(a)^	mm^(a)^	MM^(b)^	Mm^(b)^	mm^(b)^	OR^(g)^(CI)^(h)^	p-value	OR(CI)	p-value	OR(CI)	p-value	OR(CI)	p-value
rs10485057	**A/G**	67	11	0	623	102	3	1.03 (0.53–2.03)	0.927	n.c^(j)^.	—	1.00 (0.51–1.96)	1.000	n.c.	—
rs1799971	**A/G**	55	20	1	519	197	21	0.98 (0.57–1.68)	0.931	0.38 (0.05–2.91)	0.352	0.91 (0.54–1.54)	0.726	0.38 (0.05–2.91)	0.354
rs2075572	**C/G**	30	35	13	296	319	125	1.12 (0.67–1.88)	0.660	1.09 (0.55–2.17)	0.807	1.11 (0.69–1.80)	0.662	1.02 (0.55–1.92)	0.939
rs3823010	**G/A**	51	26	0	528	185	10	1.41 (0.85–2.33)	0.181	n.c.	-	1.33 (0.81–2.20)	0.264	n.c.	-
rs4870266	**G/A**	59	16	1	611	117	7	1.50 (0.83–2.71)	0.180	1.49 (0.18–12.48)	0.713	1.50 (0.84–2.67)	0.170	1.38 (0.17–11.54)	0.764
rs510769	**C/T**	40	36	0	447	252	36	1.60 (0.99–2.58)	0.055	n.c.	-	1.40 (0.87–2.26)	0.165	n.c.	-
rs540825	**T/A**	45	27	6	454	232	47	1.18 (0.71–1.96)	0.511	1.35 (0.54–3.36)	0.515	1.21 (0.75–1.95)	0.428	1.27 (0.52–3.10)	0.595
rs610231	**A/G**	54	20	1	529	171	24	1.20 (0.69–2.07)	0.517	0.44 (0.06–3.33)	0.426	1.11 (0.65–1.89)	0.711	0.42 (0.06–3.16)	0.399
rs675026	**G/A**	37	31	10	373	290	77	1.11 (0.67–1.84)	0.682	1.38 (0.65–2.91)	0.398	1.17 (0.73–1.87)	0.521	1.32 (0.65–2.68)	0.448
rs6923231	**G/A**	65	12	0	638	95	3	1.26 (0.65–2.42)	0.493	n.c.	-	1.22 (0.63–2.34)	0.557	n.c.	-
rs9322446	**G/A**	65	12	0	591	137	9	0.79 (0.41–1.51)	0.476	n.c.	-	0.74 (0.39–1.40)	0.353	n.c.	-

ABC SCORE > 0 includes newborns who do not respond to non-pharmacological analgesic treatment:

MM(a): homozygotes for the most common allele; Mm(a): heterozygotes; mm(a): homozygotes for the minor frequency allele.

ABC SCORE = 0 contains newborns who respond positively to non-pharmacological analgesic therapy:

MM(b): homozygotes for the most common allele; Mm(b): heterozygotes; mm(b): homozygotes for the minor frequency allele.

^(a)^genetic model that compares Mm vs MM (reference).

^(b)^genetic model that compares mm vs MM (reference).

^(c)^genetic model that compares Mm and mm vs MM (reference).

^(d)^genetic model that compares mm vs MM and Mm (reference).

^(e)^OR identifies the Odds Ratio.

^(f)^CI represents the Confidence Interval.

^(g)^n.c. means not calculated, due to the rarity of the minor allele.

^(h)^Analyses were adjusted for operator.

**Table 3:** Regression analysis between *OPRM1* polymorphisms and ABC SCORE > 0 corrected by gestational age.

SNP	Alleles	Codominant- heterozygous^(a)^		Codominant- Recessive^(b)^		Dominant^(c)^		Recessive^(d)^	
		Coeff^(e)^(CI) ^(f)^	p-value	Coeff(CI)	p-value	Coeff(CI)	p-value	Coeff(CI)	p-value
rs10485057	**A/G**	−0.05(−1.08–0.97)	0.923	0.59(−2.66–3.85)	0.721	0.002(−0.99–0.99)	0.996	0.60(−2.63–3.83)	0.715
rs1799971	**A/G**	0.11(−0.63–0.85)	0.772	3.18(0.13–6.23)	0.041	0.24(−0.50–0.98)	0.528	3.15(0.12–6.18)	0.041
rs2075572	**C/G**	−0.25(−1.00–0.51)	0.526	0.03(−0.99–1.05)	0.955	−0.18(−0.89–0.54)	0.628	0.17(−0.75–1.09)	0.713
rs3823010	**G/A**	−0.003(−0.73–0.72)	0.993	n.c.^(g)^	—	−0.003(−0.73–0.72)	0.993	n.c.	—
rs4870266	**G/A**	−0.24(−1.05–0.57)	0.560	−2.23(−5.29–0.83)	0.154	−0.35(−1.14–0.45)	0.392	−2.18(−5.22–0.87)	0.161
rs510769	**C/T**	−0.28(−0.96–0.40)	0.425	n.c.	—	−0.28(−0.96–0.40)	0.425	n.c.	—
rs540825	**T/A**	−0.14(−0.88–0.60)	0.707	0.80(−0.47–2.07)	0.218	0.03(−0.66–0.73)	0.923	0.86(−0.37–2.09)	0.172
rs610231	**A/G**	−0.15(−0.95–0.64)	0.705	−0.99(−4.20–2.22)	0.547	−0.19(−0.97–0.59)	0.634	−0.94(−4.13–2.24)	0.562
rs675026	**G/A**	−0.04(−0.78–0.70)	0.912	0.39(−0.69–1.46)	0.480	0.06(−0.63–0.75)	0.866	0.41(−0.60–1.42)	0.427
rs6923231	**G/A**	−0.04(−1.00–0.91)	0.931	n.c.	—	−0.04(−1.00–0.91)	0.931	n.c.	—
rs9322446	**G/A**	0.27(−0.68–1.21)	0.580	n.c.	—	0.27(-0.68–1.21)	0.580	n.c.	—

^(a)^genetic model that compares heterozygotes vs homozygotes for the most common allele (reference).

^(b)^genetic model that compares homozygotes for the minor frequency allele vs homozygotes for the most common allele (reference).

^(c)^genetic model that compares heterozygotes and homozygotes for the minor frequency allele vs homozygotes for the most common allele (reference).

^(d)^genetic model that compares homozygotes for the minor frequency allele vs heterozygotes and homozygotes for the most common allele (reference).

^(e)^Coeff. identifies linear regression coefficient.

^(f)^CI represents the Confidence Interval.

^(g)^n.c. means not calculated, due to the rarity of the minor allele.

### Possible functional effects

Haploreg and RegulomeDB did not show any potential functional effect for the SNPs taken into consideration and the GTEx database did not suggest any eQTLs for the rs1799971-SNP.

## Discussion

In this study we have enrolled more than 1000 newborns to investigate whether genetic variability and anthropometric and lifestyle factors could influence non-pharmacologic analgesic treatment in newborns. This sample size makes it one of the largest studies on newborn genetics with the addition of meticulously collected information on anthropometric and life style factors. The efficacy of the non-pharmacologic treatment was very good affecting 966 (92%) out of 1054 individuals.

We observed an association between feeding type and analgesic efficacy of the non-pharmacologic treatment. The association was significant in the crude model and with adjustment. The trend showed a clear association between natural human milk and increased chance of effective analgesic treatment (p = 0.005). A possible explanation of this association might reside in the fact that breast feeding could have a prolonged soothing effect on newborns, decreasing their anxiety and increasing the sugar effect. The pain relief effect of breast milk could also be explained by the higher concentration of tryptophan compared to formula milk. As suggested by Heine, tryptophan is a precursor of melatonin, which can increase beta endorphin production regulating appetite, satisfaction and pain perception^31^. The relative relevance of the two different aspects could not be weighted because all patients that received human milk were breastfed and did not receive expressed breast milk or human milk from donors.

From a genetic point of view, we observed that in the logistic regression (ABC score > 0 *vs* ABC score = 0) rs510769 was close to the conventional threshold for statistical significance (p = 0.055) and that in the glm model (ABC score from 1 to 6) the carriers of the G allele of the rs1799971 showed the least benefit from the analgesic treatment. *OPRM1*-rs510769 is an intronic variant that has been investigated in relation to various human traits, such as onset of side effects in patients during a methadone maintenance treatment (MMT)^32^, smoking behavior in MMT^33^, susceptibility to heroin addiction^34^ and amphetamine-induced euphoria^35^. However, there are no functional evidences in the literature for this SNP; in addition, the results from the bioinformatic tools we used are inconclusive. Therefore, it is not easy to infer a mechanistic relation between the SNP and analgesic treatment.

On the other hand, rs1799971 (A118G) is the most studied variant in the *OPRM1* gene and there are overwhelming evidence, spanned among a decade, supporting its role in a variety of human phenotypes including pain, analgesia and drug tolerance^18–21,24,25,33,36–39^. This polymorphism is a missense variant with an A to G nucleotide change that leads to an amino-acid substitution (Asn40Asp) at a putative N-glycosylation site in the extracellular receptor region. The majority of the studies support an increased pain sensitivity and worse response to pain relief therapy in individuals with the GG genotype compared to the other genotypes^18–21,24,25^. Changes from a basic amino acid to an acid amino acid in the OPRM1 receptor could alter its ability to bind ligands and could explain the altered effectiveness of the protein. In agreement with what suggested by the literature, we observed a tendency for GG homozygous to display less affective analgesic efficacy, even though in a subgroup analysis. This difference may be explained by the fact that pain relief treatment is a complex experience that is mediated by several variables. Indeed, a single SNP is unlikely to predict the ability to respond to the therapy, also considering the relative small size of non-responders in our population. However, our results suggest, even though with a weak statistical association, that among the individuals that do not respond to the therapy the intensity of the score could be mediated by the genotype of the rs1799971 (A118G) variant. This result should be interpreted with caution since it comes from a subgroup analysis, and therefore from a small number of individuals; in light of the multiple tests that we performed it could be statistical fluctuation. However, our findings are in line with what has been repeatedly observed for adults, i.e. the GG genotype of the rs1799971 (A118G) SNP associated with less effectiveness of pain reducing treatments.

In conclusion, this study highlights that the type of milk seems to be associated with newborn pain treatment response and also suggests a possible association between the missense variant rs1799971 (A118G) and pain reduction in newborns. These findings if further replicated could represent an important step in evaluating the possibility of a personalized analgesia in newborns.

## Supplementary information

Supplementary tables 1–6.

## Data Availability

The data for this work will be made available to researchers who submit a reasonable and detailed request to the corresponding author, conditional to approval of the Ethics Commission of the of the Meyer Children Hospital of Florence. Data will be stripped from all information allowing identification of study participants.
