# Supplementary material for: Role of OPRM1, clinical and anthropometric variants in neonatal pain reduction
Source: Sci Rep. 2020 Apr 27;10:7091. doi: 10.1038/s41598-020-63790-2 (PMC7184594; doi:10.1038/s41598-020-63790-2)
Supplement: Supplementary file 1 — Supplementary tables 1–6. [file 41598_2020_63790_MOESM1_ESM.docx]

**Role of *OPRM1*, clinical and anthropometric variants in neonatal pain reduction**

Ilaria Erbi^1^, Massimiliano Ciantelli^2^, Riccardo Farinella^1^, Cristina Tuoni^2^, Manuel Gentiluomo^1^, Francesca Moscuzza^2^, Cosmeri Rizzato^3^, Alice Bedini^2^, Maddalena Faraoni^2^, Stefano Giusfredi^1^, Arianna Tavanti^1^, Paolo Ghirri^2^, Daniele Campa^1^

1 Department of Biology, University of Pisa, Pisa

2 Division of Neonatology – Santa Chiara Hospital - Pisa

3 Department of Translation Research and of New Technologies in Medicine and Surgery, University of Pisa, Pisa

**Corresponding author:**

Daniele Campa

Department of Biology,

Pisa University, Pisa Italy

daniele.campa@unipi.it

Tel:0039-050-2211510

Fax:0039-050-2211527

institutional URL: https://www.unipi.it/

**Supplementary table 1. Logistic analysis of anthropometric, clinical and lifestyle variables and ABC score adjusted for operator.**

|  | **Logistic analysis**  **corrected for procedure's executor** | |
| --- | --- | --- |
| **Covariates** | **OR^(a)^(CI)^(b)^** | **p-value** |
| Gender_(m/f) | 0.9(0.56-1.43) | 0.651 |
| Gestational age_(weeks) | 0.96(0.79-1.17) | 0.718 |
| Feeding type_(maternal/ mixed/ artificial) | 1.64(1.11-2.43) | 0.014 |
| Mode of birth_(vaginal delivery/ cesarean section) | 0.98(0.61-1.56) | 0.926 |
| Maternal age_(years) | 1.03(0.98-1.08) | 0.204 |
| Spinal anesthesia_(yes/no) | 1.1(0.66-1.81) | 0.720 |
| Epidural anesthesia_(yes/no) | 0.96(0.52-1.77) | 0.884 |
| General anesthesia_(yes/no) | 1.51(0.18-12.36) | 0.701 |
| Birth weight_(gramms) | 0.99(0.99-1) | 0.056 |
| Maternal gestational diabetes_(yes/no) | 1.31(0.71-2.44) | 0.386 |
| Mother's smoke_(yes/no) | 1.7(0.69-4.22) | 0.250 |

**Legend:**

1. OR identifies the Odds Ratio.
2. CI represents the Confidence Interval

**Supplementary table 2. Regression analysis of anthropometric, clinical and lifestyle variables and ABC score>0**

| **Covariates** | **Coeff.^(a)^(CI) ^(b)^** | **p-value** |
| --- | --- | --- |
| Gender_(m/f) | -0.02(-0.71-0.66) | 0.949 |
| Gestational age_(weeks) | 0.27(-0.02-0.57) | 0.067 |
| Procedure's executor_n | 0.03(0-0.05) | 0.050 |
| Feeding type_(maternal/ mixed/ artificial) | -0.17(-0.74-0.41) | 0.573 |
| Mode of birth_(vaginal delivery/ cesarean section) | -0.39(-1.07-0.29) | 0.262 |
| Maternal age_(years) | -0.02(-0.09-0.06) | 0.701 |
| Spinal anesthesia_(yes/no) | -0.18(-0.9-0.54) | 0.624 |
| Epidural anesthesia_(yes/no) | 0.59(-0.33-1.52) | 0.209 |
| General anesthesia_(yes/no) | -1.07(-4.16-2.03) | 0.498 |
| Birth weight_(gramms) | 0.0005(-0.0003-0.0013) | 0.248 |
| Maternal gestational diabetes_(yes/no) | -0.36(-1.27-0.54) | 0.429 |
| Mother's smoke_(yes/no) | -0.04(-1.4-1.32) | 0.956 |

**Legend:**

1. Coeff identifies linear regression coefficient
2. CI represents the Confidence Interval

**Supplementary Table 3. Association between *OPRM1* polymorphisms and ABC score (ABC score>0 *vs* ABC score =0).**

| **SNP** | **ALLELES** | **ABC SCORE>0^(a)^** | | | **ABC SCORE=0^(b)^** | | | **Codominant-**  **heterozygous^(c)^** | | **Codominant-**  **Recessive ^(d)^** | | **Dominant^(e)^** | | **Recessive ^(f)^** | |
| --- | --- | --- | --- | --- | --- | --- | --- | --- | --- | --- | --- | --- | --- | --- | --- |
|  |  | **MM^(a)^** | **Mm^(a)^** | **Mm^(a)^** | **MM^(b)^** | **Mm^(b)^** | **Mm^(b)^** | **OR^(g)^(CI)^(h)^** | **p-value** | **OR(CI)** | **p-value** | **OR(CI)** | **p-value** | **OR(CI)** | **p-value** |
| **rs10485057** | **A/G** | 75 | 11 | 1 | 793 | 128 | 5 | 0.91 (0.47-1.76) | 0.776 | 2.11 (0.24-18.34) | 0.497 | 0.95 (0.5-1.8) | 0.885 | 2.14 (0.25-18.54) | 0.489 |
| **rs1799971** | **A/G** | 61 | 23 | 1 | 668 | 243 | 25 | 1.04 (0.63-1.71) | 0.889 | 0.44 (0.06-3.29) | 0.422 | 0.98 (0.6-1.61) | 0.938 | 0.43 (0.06-3.24) | 0.416 |
| **rs2075572** | **C/G** | 34 | 38 | 15 | 364 | 424 | 151 | 0.96 (0.59-1.56) | 0.867 | 1.06 (0.56-2.01) | 0.850 | 0.99 (0.63-1.55) | 0.954 | 1.09 (0.61-1.95) | 0.779 |
| **rs3823010** | **G/A** | 59 | 27 | 0 | 680 | 224 | 17 | 1.39 (0.86-2.24) | 0.179 | n.c.^(i)^ | - | 1.29 (0.8-2.08) | 0.295 | n.c. | - |
| **rs4870266** | **G/A** | 66 | 18 | 1 | 773 | 151 | 8 | 1.4 (0.81-2.42) | 0.234 | 1.46 (0.18-11.88) | 0.721 | 1.4 (0.82-2.4) | 0.221 | 1.38 (0.17-11.13) | 0.765 |
| **rs510769** | **C/T** | 48 | 37 | 0 | 567 | 318 | 46 | 1.37 (0.88-2.16) | 0.166 | n.c. | - | 1.2 (0.77-1.88) | 0.424 | n.c. | - |
| **rs540825** | **T/A** | 50 | 30 | 7 | 570 | 304 | 58 | 1.12 (0.7-1.81) | 0.626 | 1.38 (0.6-3.17) | 0.454 | 1.17 (0.75-1.82) | 0.501 | 1.32 (0.58-2.98) | 0.507 |
| **rs610231** | **A/G** | 61 | 22 | 1 | 664 | 225 | 31 | 1.06 (0.64-1.77) | 0.811 | 0.35 (0.05-2.62) | 0.307 | 0.98 (0.59-1.61) | 0.931 | 0.35 (0.05-2.56) | 0.299 |
| **rs675026** | **G/A** | 41 | 34 | 12 | 468 | 373 | 96 | 1.04 (0.65-1.67) | 0.870 | 1.43 (0.72-2.82) | 0.305 | 1.12 (0.72-1.74) | 0.615 | 1.4 (0.74-2.67) | 0.305 |
| **rs6923231** | **G/A** | 73 | 13 | 0 | 813 | 117 | 5 | 1.24 (0.66-2.3) | 0.501 | n.c. | - | 1.19 (0.64-2.21) | 0.588 | n.c. | - |
| **rs9322446** | **G/A** | 73 | 13 | 0 | 742 | 183 | 11 | 0.72 (0.39-1.33) | 0.297 | n.c. | - | 0.68 (0.37-1.25) | 0.218 | n.c. | - |

**Legend:**

1. ABC SCORE>0 includes newborns who do not respond to non-pharmacological analgesic treatment:

MM^(a)^: homozygotes for the most common allele; Mm^(a)^: heterozygotes; mm^(a)^: homozygotes for the minor frequency allele.

1. ABC SCORE =0 contains newborns who respond positively to non-pharmacological analgesic therapy:

MM^(b)^: homozygotes for the most common allele; Mm^(b)^: heterozygotes; mm^(b)^: homozygotes for the minor frequency allele.

1. genetic model that compares Mm vs MM (reference)
2. genetic model that compares mm vs MM (reference)
3. genetic model that compares Mm and mm vs MM (reference)
4. genetic model that compares mm vs MM and Mm (reference)
5. OR identifies the Odds Ratio.
6. CI represents the Confidence Interval
7. n.c. means not calculated, due to the rarity of the minor allele.

**Supplementary table 4. Regression analysis between *OPRM1* polymorphisms and ABC SCORE>0 crude analysis*.***

| **SNP** | **Alleles** | **Codominant-**  **heterozygous^(a)^** | | **Codominant-**  **Recessive^(b)^** | | **Dominant^(c)^** | | **Recessive^(d)^** | |
| --- | --- | --- | --- | --- | --- | --- | --- | --- | --- |
|  |  | **Coeff^(e)^(CI) ^(f)^** | **p-value** | **Coeff(CI)** | **p-value** | **Coeff(CI)** | **p-value** | **Coeff(CI)** | **p-value** |
| rs10485057 | A/G | -0.14 (-1.18-0.89) | 0.785 | -0.05(-3.28-3.17) | 0.974 | -0.14 (-1.13-0.85) | 0.787 | -0.03 (-3.24-3.17) | 0.983 |
| rs1799971 | A/G | 0.15 (-0.61-0.91) | 0.694 | 3.07(-0.06-6.19) | 0.055 | 0.27 (-0.48-1.03) | 0.479 | 3.02 (-0.08-6.13) | 0.056 |
| rs2075572 | C/G | -0.37 (-1.12-0.39) | 0.340 | -0.24(-1.22-0.75) | 0.641 | -0.33 (-1.03-0.37) | 0.354 | -0.04 (-0.95-0.86) | 0.928 |
| rs3823010 | G/A | -0.02 (-0.76-0.72) | 0.964 | n.c.^(g)^ | - | -0.02 (-0.76-0.72) | 0.964 | n.c. | - |
| rs4870266 | G/A | -0.33 (-1.16-0.5) | 0.440 | -2.11(-5.26-1.05) | 0.191 | -0.42 (-1.24-0.39) | 0.311 | -2.04 (-5.18-1.11) | 0.204 |
| rs510769 | C/T | -0.25 (-0.94-0.44) | 0.471 | n.c. | - | -0.25 (-0.94-0.44) | 0.471 | n.c. | - |
| rs540825 | T/A | -0.28 (-1.01-0.45) | 0.454 | 0.63(-0.65-1.91) | 0.331 | -0.11 (-0.8-0.58) | 0.761 | 0.74 (-0.51-1.99) | 0.245 |
| rs610231 | A/G | -0.23 (-1.03-0.56) | 0.565 | -1.1(-4.34-2.14) | 0.507 | -0.27 (-1.06-0.51) | 0.496 | -1.04 (-4.26-2.19) | 0.528 |
| rs675026 | G/A | -0.13 (-0.87-0.61) | 0.728 | 0.09(-0.96-1.14) | 0.862 | -0.07 (-0.76-0.61) | 0.834 | 0.15 (-0.84-1.14) | 0.762 |
| rs6923231 | G/A | -0.19 (-1.15-0.76) | 0.689 | n.c. | - | -0.19 (-1.15-0.76) | 0.689 | n.c. | - |
| rs9322446 | G/A | 0.36 (-0.58-1.31) | 0.451 | n.c. | - | 0.36 (-0.58-1.31) | 0.451 | n.c. | - |

**Legend:**

1. genetic model that compares heterozygotes vs homozygotes for the most common allele (reference)
2. genetic model that compares homozygotes for the minor frequency allele vs homozygotes for the most common allele (reference)
3. genetic model that compares heterozygotes and homozygotes for the minor frequency allele vs homozygotes for the most common allele (reference)
4. genetic model that compares homozygotes for the minor frequency allele vs heterozygotes and homozygotes for the most common allele (reference)
5. Coeff. identifies linear regression coefficient
6. CI represents the Confidence Interval
7. n.c. means not calculated, due to the rarity of the minor allele.

**Supplementary table 5. Regression analysis between *OPRM1* polymorphisms and ABC SCORE>0 corrected by operator*.***

| **SNP** | **Alleles** | **Codominant-**  **heterozygous^(a)^** | | **Codominant-**  **Recessive^(b)^** | | **Dominant^(c)^** | | **Recessive^(d)^** | |
| --- | --- | --- | --- | --- | --- | --- | --- | --- | --- |
|  |  | **Coeff^(e)^(CI) ^(f)^** | **p-value** | **Coeff(CI)** | **p-value** | **Coeff(CI)** | **p-value** | **Coeff(CI)** | **p-value** |
| rs10485057 | A/G | -0.17(-1.18-0.84) | 0,739 | n.c.^(g)^ | - | -0.17(-1.18-0.84) | 0,739 | n.c. | - |
| rs1799971 | A/G | 0.09(-0.7-0.88) | 0,827 | 2.81(-0.24-5.86) | 0,071 | 0.22(-0.56-1.01) | 0,576 | 2.79(-0.23-5.82) | 0,070 |
| rs2075572 | C/G | -0.42(-1.19-0.35) | 0,283 | -0.47(-1.5-0.55) | 0,364 | -0.44(-1.15-0.28) | 0,232 | -0.25(-1.19-0.69) | 0,604 |
| rs3823010 | G/A | 0.02(-0.73-0.76) | 0,968 | n.c. | - | 0.02(-0.73-0.76) | 0,968 | n.c. | - |
| rs4870266 | G/A | -0.2(-1.06-0.65) | 0,642 | -1.55(-4.66-1.57) | 0,331 | -0.28(-1.12-0.56) | 0,515 | -1.5(-4.59-1.59) | 0,343 |
| rs510769 | C/T | -0.19(-0.89-0.52) | 0,601 | n.c. | - | -0.19(-0.89-0.52) | 0,601 | n.c. | - |
| rs540825 | T/A | -0.28(-1.03-0.48) | 0,471 | 0.08(-1.28-1.44) | 0,911 | -0.21(-0.92-0.49) | 0,554 | 0.18(-1.15-1.5) | 0,793 |
| rs610231 | A/G | -0.58(-1.38-0.23) | 0,161 | -0.86(-3.98-2.26) | 0,589 | -0.59(-1.38-0.2) | 0,141 | -0.71(-3.85-2.42) | 0,657 |
| rs675026 | G/A | -0.07(-0.83-0.69) | 0,853 | -0.35(-1.46-0.77) | 0,545 | -0.14(-0.84-0.56) | 0,697 | -0.31(-1.38-0.75) | 0,562 |
| rs6923231 | G/A | -0.22(-1.19-0.75) | 0,650 | n.c. | - | -0.22(-1.19-0.75) | 0,650 | n.c. | - |
| rs9322446 | G/A | 0.43(-0.52-1.37) | 0,377 | n.c. | - | 0.43(-0.52-1.37) | 0,377 | n.c. | - |

**Legend:**

1. genetic model that compares heterozygotes vs homozygotes for the most common allele (reference)
2. genetic model that compares homozygotes for the minor frequency allele vs homozygotes for the most common allele (reference)
3. genetic model that compares heterozygotes and homozygotes for the minor frequency allele vs homozygotes for the most common allele (reference)
4. genetic model that compares homozygotes for the minor frequency allele vs heterozygotes and homozygotes for the most common allele (reference)
5. Coeff. identifies linear regression coefficient
6. CI represents the Confidence Interval
7. n.c. means not calculated, due to the rarity of the minor allele.

**Supplementary table 6. Regression analysis between *OPRM1* polymorphisms and ABC SCORE>0 corrected by gestational age and operator*.***

| **SNP** | **Alleles** | **Codominant-**  **heterozygous^(a)^** | | **Codominant-**  **Recessive^(b)^** | | **Dominant^(c)^** | | **Recessive^(d)^** | |
| --- | --- | --- | --- | --- | --- | --- | --- | --- | --- |
|  |  | **Coeff^(e)^(CI) ^(f)^** | **p-value** | **Coeff(CI)** | **p-value** | **Coeff(CI)** | **p-value** | **Coeff(CI)** | **p-value** |
| rs10485057 | A/G | -0.1(-1.09-0.89) | 0.847 | n.c. ^(g)^ | - | -0.1(-1.09-0.89) | 0.847 | n.c. | - |
| rs1799971 | A/G | 0.1(-0.67-0.86) | 0.807 | 2.84(-0.11-5.79) | 0.059 | 0.23(-0.53-0.99) | 0.550 | 2.82(-0.11-5.74) | 0.059 |
| rs2075572 | C/G | -0.31(-1.07-0.46) | 0.429 | -0.18(-1.24-0.88) | 0.745 | -0.28(-1-0.45) | 0.452 | 0.01(-0.95-0.96) | 0.989 |
| rs3823010 | G/A | -0.02(-0.75-0.7) | 0.948 | n.c. | - | -0.02(-0.75-0.7) | 0.948 | n.c. | - |
| rs4870266 | G/A | -0.18(-1-0.64) | 0.672 | -1.47(-4.45-1.5) | 0.332 | -0.25(-1.05-0.55) | 0.543 | -1.43(-4.38-1.52) | 0.342 |
| rs510769 | C/T | -0.26(-0.95-0.43) | 0.465 | n.c. | - | -0.26(-0.95-0.43) | 0.465 | n.c. | - |
| rs540825 | T/A | -0.11(-0.87-0.65) | 0.781 | 0.3(-1.05-1.66) | 0.660 | -0.04(-0.75-0.68) | 0.923 | 0.35(-0.96-1.66) | 0.602 |
| rs610231 | A/G | -0.48(-1.28-0.33) | 0.246 | -0.67(-3.76-2.43) | 0.673 | -0.49(-1.27-0.3) | 0.226 | -0.53(-3.62-2.57) | 0.739 |
| rs675026 | G/A | 0.02(-0.73-0.77) | 0.963 | -0.08(-1.21-1.05) | 0.889 | 0.005(-0.7-0.7) | 0.990 | -0.09(-1.16-0.98) | 0.870 |
| rs6923231 | G/A | -0.12(-1.08-0.83) | 0.799 | n.c. | - | -0.12(-1.08-0.83) | 0.799 | n.c. | - |
| rs9322446 | G/A | 0.31(-0.64-1.26) | 0.520 | n.c. | - | 0.31(-0.64-1.26) | 0.520 | n.c. | - |

**Legend:**

1. genetic model that compares heterozygotes vs homozygotes for the most common allele (reference)
2. genetic model that compares homozygotes for the minor frequency allele vs homozygotes for the most common allele (reference)
3. genetic model that compares heterozygotes and homozygotes for the minor frequency allele vs homozygotes for the most common allele (reference)
4. genetic model that compares homozygotes for the minor frequency allele vs heterozygotes and homozygotes for the most common allele (reference)
5. Coeff. identifies linear regression coefficient
6. CI represents the Confidence Interval
7. n.c. means not calculated, due to the rarity of the minor allele.
